# Supplementary material for: The SIAH2-NRF1 axis spatially regulates tumor microenvironment remodeling for tumor progression
Source: Nat Commun. 2019 Mar 4;10:1034. doi: 10.1038/s41467-019-08618-y (PMC6399320; doi:10.1038/s41467-019-08618-y)
Supplement: Supplementary file 1 — Supplementary Information [file 41467_2019_8618_MOESM1_ESM.pdf]

Supplementary Information

The SIAH2-NRF1 axis spatially regulates tumor microenvironment remodeling for tumor progression

Ma et al.

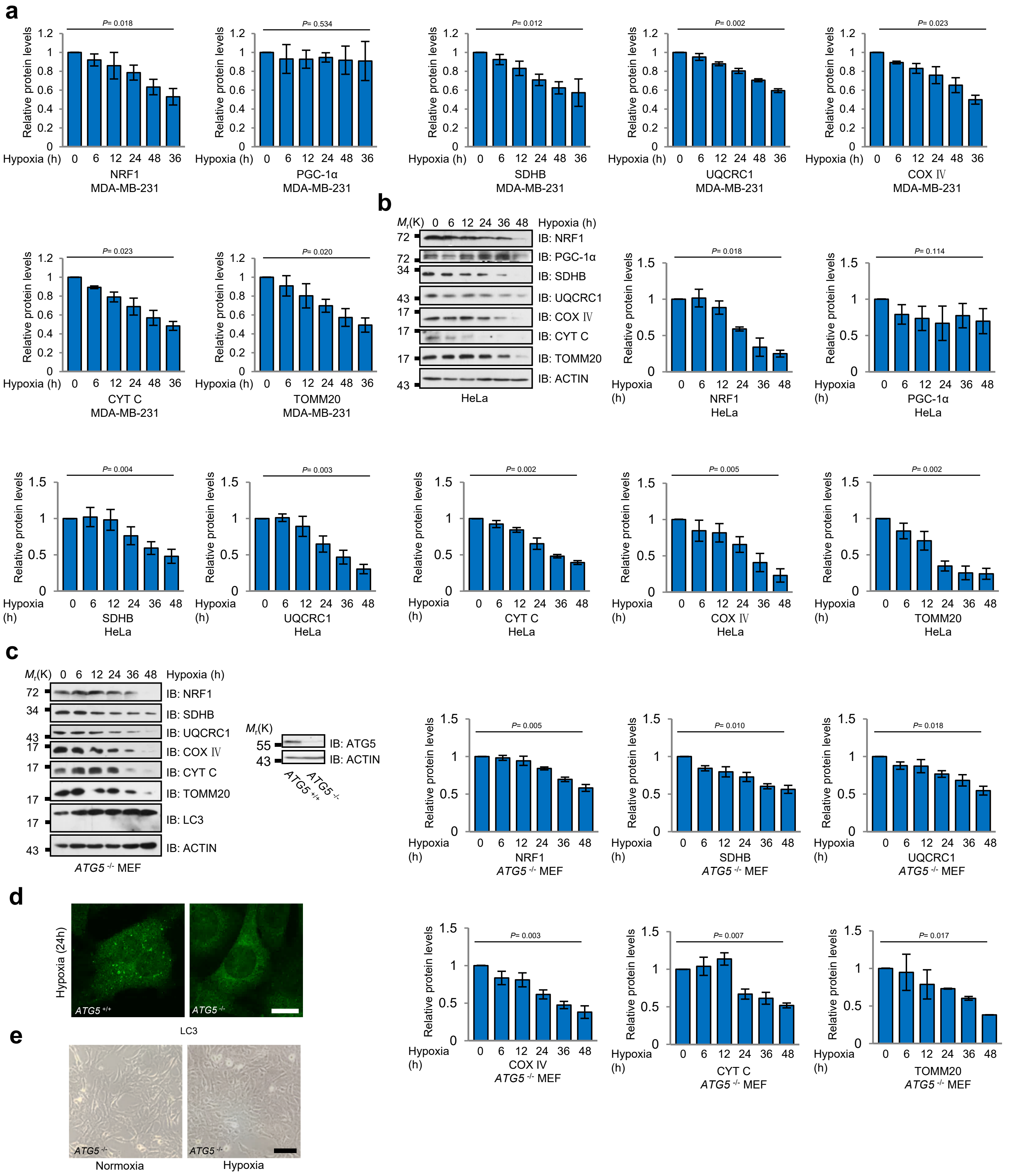

Supplementary Figure 1 Hypoxia reduces nuclear-encoded mitochondrial proteins in multiple cell lines. (**a**) Statistical analysis of data from Figure 2a. (**b**) HeLa cells cultured under hypoxia were analyzed by immunoblotting with the indicated antibodies at the indicated time points. Immunoblot results were quantified by densitometry, and the data were compared statistically, as shown in the graphs. (**c**) *ATG5*$^{-/-}$ MEFs cultured under normoxia or hypoxia were analyzed by immunoblotting using the indicated antibodies at the indicated time points. Results were quantified and compared as in (b). (**d**) Confocal images of *ATG5*$^{+/+}$ and *ATG5*$^{-/-}$ MEFs cultured under hypoxia for 36 h and stained with anti-LC3 antibodies. Scale bars, 15 μm. (**e**) Bright-field images of *ATG5*$^{-/-}$ MEFs cultured under normoxia and hypoxia for 36 h. Scale bars, 100 μm. For all panels, error bars indicate s.d., n = 3 biological replicates. One-way ANOVA was used to compare data.

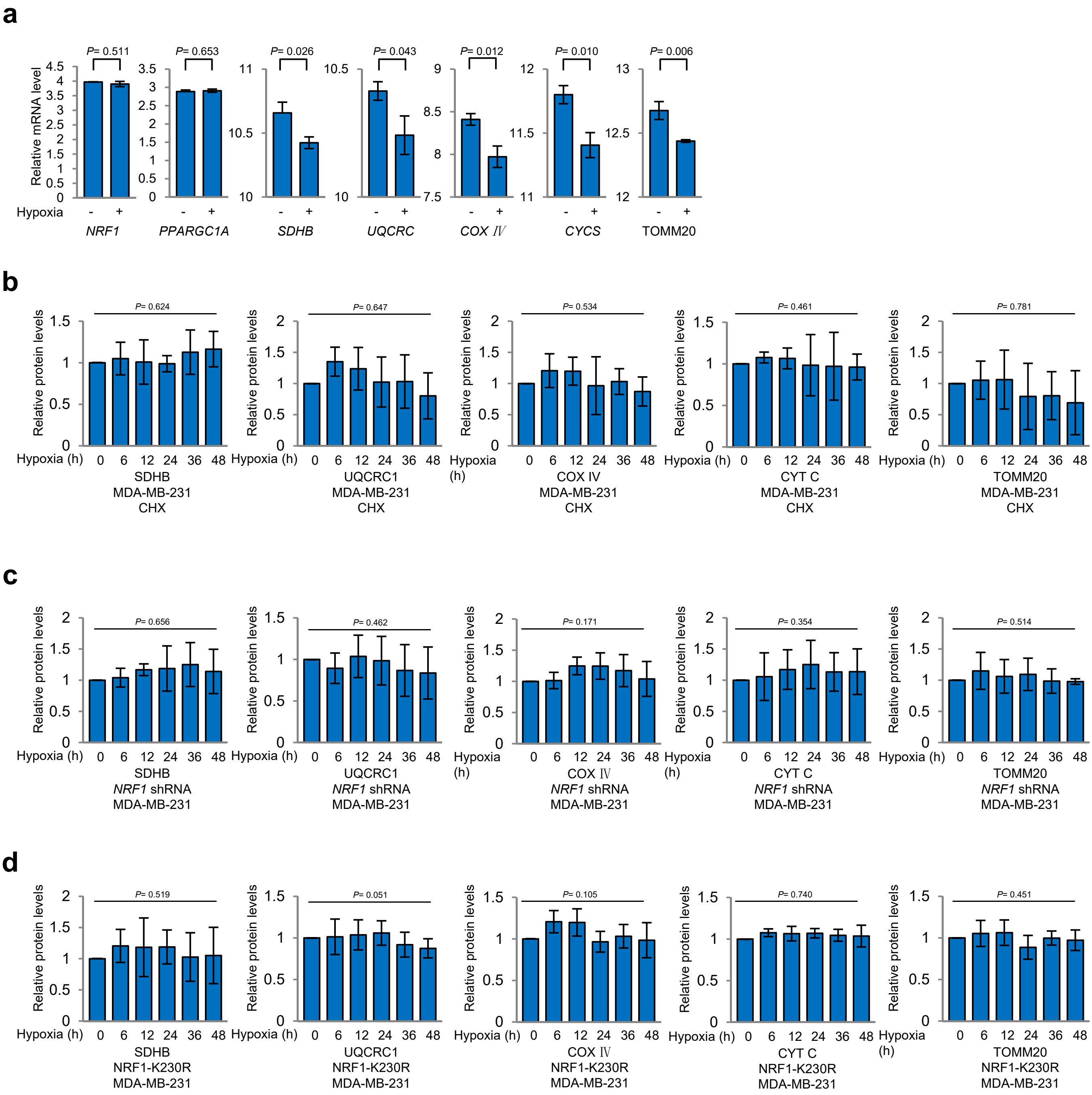

Supplementary Figure 2 Hypoxia inhibits nuclear-encoded mitochondrial gene expression through NRF1. (**a**) Statistical analysis of the expression levels of the indicated genes in dataset GSE18494. (**b**) Statistical analysis of data from Figure 2c. (**c**) Statistical analysis of data from Figure 2d. (**d**) Statistical analysis of data from Figure 5e. For all panels, error bars indicate s.d., n = 3 biological replicates, average of n=3 technical replicates for each biological replicate was used. One-way ANOVA was used to compare data.

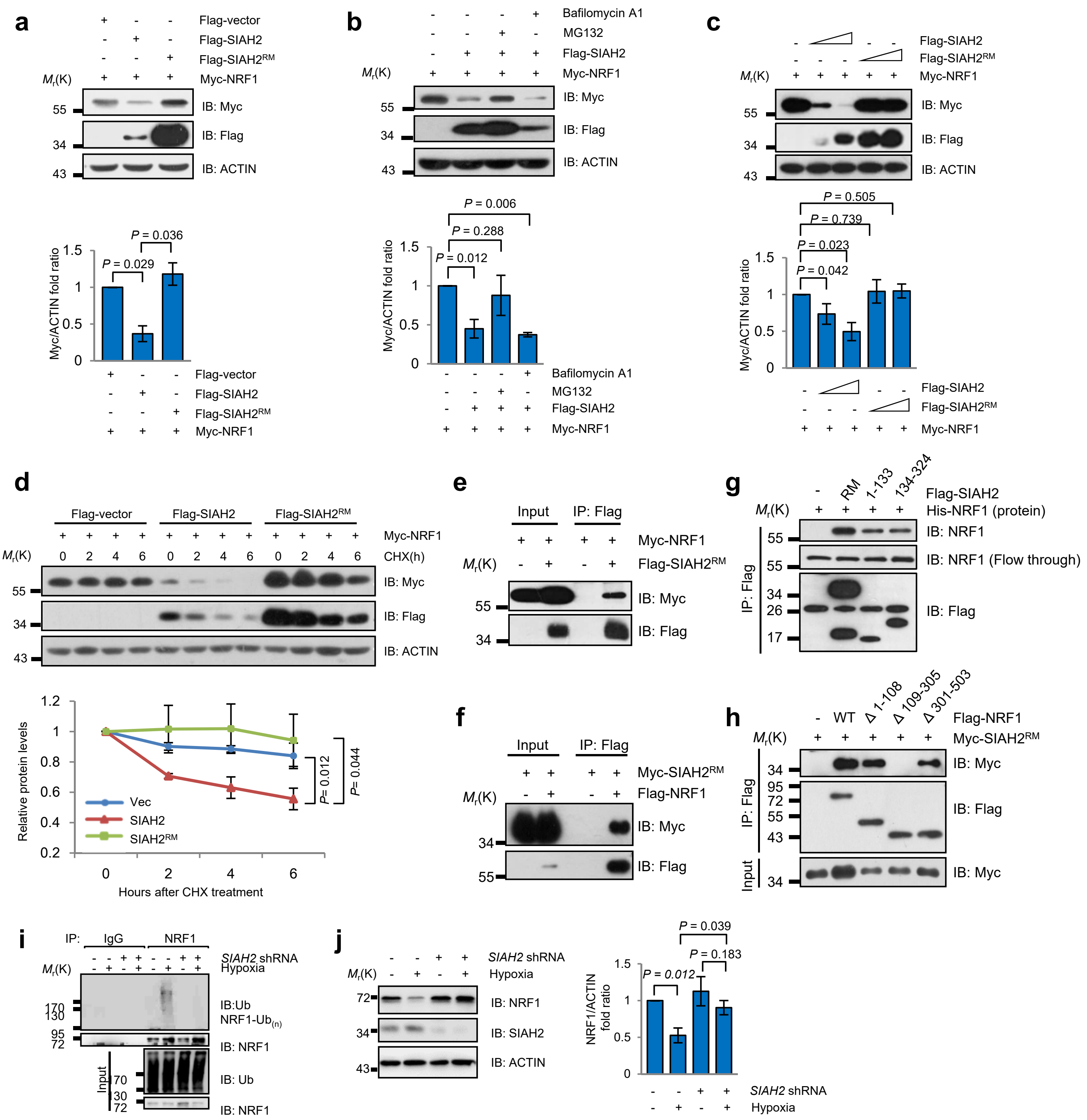

Supplementary Figure 3 SIAH2 interacts with NRF1 and negatively regulates its stability through ubiquitination. (**a**) HeLa cells transiently expressing SIAH2 or SIAH2RM (Ref. 34) (a dominant-negative mutant which lacks ubiquitin E3 ligase activity but retains the ability to bind to its substrate) together with NRF1 were collected for immunoblotting with the indicated antibodies. Quantification of NRF1 protein levels is shown below. (**b**) HeLa cells transiently expressing SIAH2 or SIAH2RM together with NRF1 for 24 h were treated with 10 μM proteasomal inhibitor MG132 or 100 nM autophagy inhibitor Bafilomycin A1 (BafA1) for an additional 6 h. Cells were collected for immunoblotting with the indicated antibodies. Quantification of NRF1 protein levels is shown below. (**c**) NRF1 degradation depends on the dosage of SIAH2, but is unaffected by SIAH2RM. Quantification of NRF1 protein levels is shown below. (**d**) The half-life of NRF1 is shortened by SIAH2, but not by SIAH2RM in cycloheximide chase assays. Quantification of NRF1 protein levels is shown below. (**e**, **f**) Co-immunoprecipitations of exogenously expressed Myc-NRF1 with Flag-SIAH2RM (**e**) and vice versa (**f**) in HEK293T cells. (**g**) Co-immunoprecipitations of exogenously expressed Myc-NRF1 with the N- or C-terminal half of Flag-SIAH2. (**h**) Dissection of the critical regions of NRF1 for SIAH2 binding through co-immunoprecipitation in HEK293T cells. (**i**) Decreased hypoxia-induced NRF1 ubiquitination by knockdown of SIAH2. (**j**) Scramble and SIAH2-knockdown MDA-MB-231 cells were cultured under normoxia or hypoxia for 36 h, and then were collected for analysis using the indicated antibodies. Quantification of NRF1 protein levels is shown on the right. For all panels, error bars indicate s.d.. For panel (a-d) and (j), n = 3 biological replicates. The two-tailed paired ratio t-test was used to compare data.

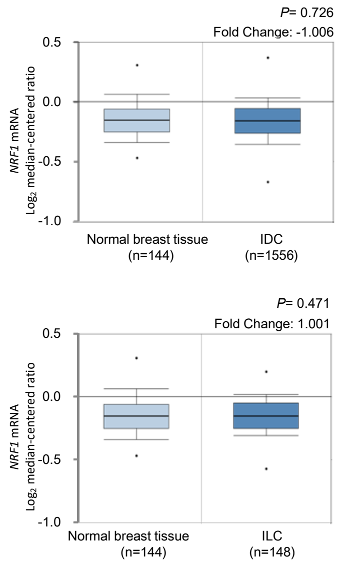

Supplementary Figure 4 Transcriptional analysis of NRF1 in breast cancer patients with invasive ductal carcinoma (IDC) or invasive lobular carcinoma (ILC) based on Oncomine.

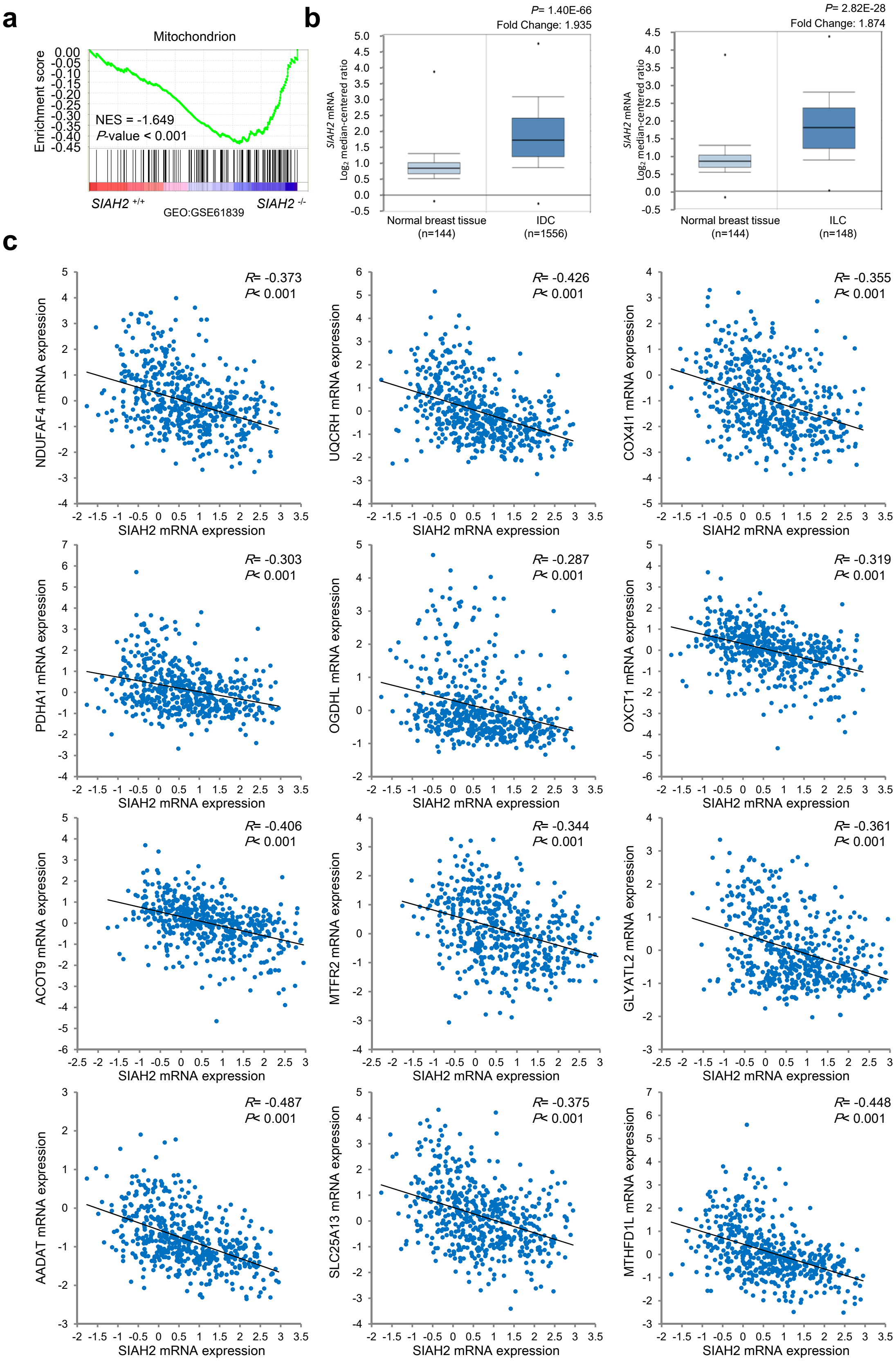

Supplementary Figure 5 SIAH2 negatively correlates with mitochondrial genes expression. (**a**) GO enrichment in mitochondrial genes in SIAH2-/- mouse tissues from dataset GSE61839, with a FDR of < 25%. (**b**) Transcriptional analysis of SIAH2 in breast cancer patients with invasive ductal carcinoma (IDC) or invasive lobular carcinoma (ILC) based on Oncomine. (**c**) Analysis of the correlations between the transcriptional levels of SIAH2 and multiple nuclear-encoded mitochondrial genes in breast cancer patients based on the Cancer Genome Atlas (TCGA) database using cBioPortal.

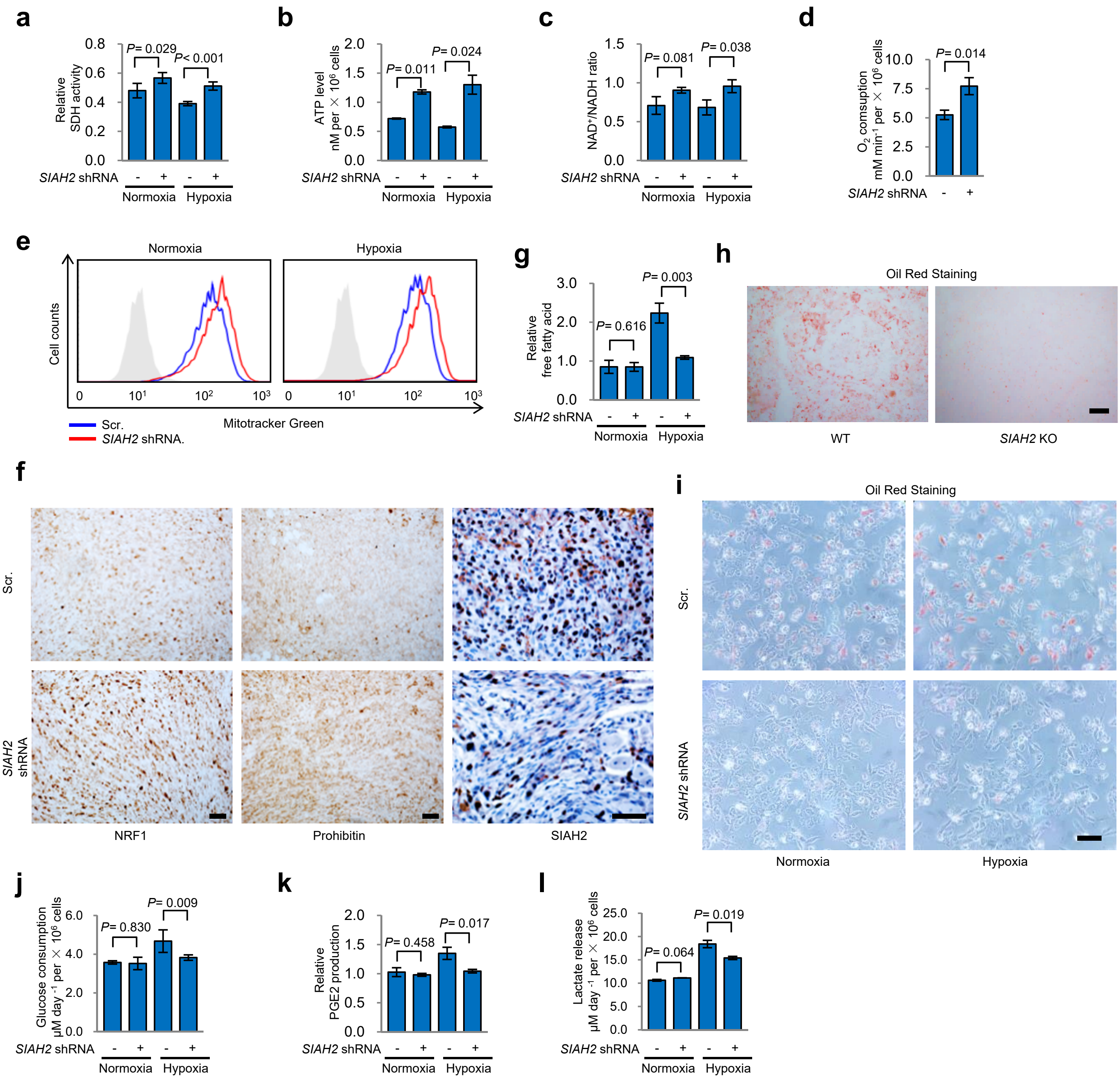

Supplementary Figure 6 SIAH2 facilitates hypoxia-induced metabolic reprogramming. (**a-c**) Wild-type and SIAH2-knockdown MDA-MB-231 cells were cultured under normoxia or hypoxia for 36 h, and then mitochondrial SDH activity (**a**), ATP levels (**b**) and NAD+/NADH ratios (**c**) were analyzed. (**d**) Oxygen consumption rate was determined in wild-type and SIAH2-knockdown MDA-MB-231 cells. (**e**) Wild-type and SIAH2-knockdown MDA-MB-231 cells were cultured under normoxia or hypoxia for 36 h, cells were stained with 100 nM MitoTracker Green and analyzed with flow cytometry. (**f**) The indicated xenograft tumor tissues were immunohistochemically analyzed by staining with the indicated antibodies. Scale bars, 50 μm. (**g**) Wild-type and SIAH2-knockdown MDA-MB-231 cells were cultured under normoxia or hypoxia for 36 h, then the concentrations of free fatty acids were analyzed. (**h**) Wild-type and SIAH2-/- xenograft tissues were stained by Oil Red. Scale bars, 50 μm. (**i**) Wild-type and SIAH2-knockdown MDA-MB-231 cells were treated with 0.5 mM palmitic acid and cultured under normoxia or hypoxia for 24 h. Cells were stained by Oil Red. Scale bars, 100 μm. (**j-l**) Wild-type and SIAH2-knockdown MDA-MB-231 cells were cultured under normoxia or hypoxia for 36 h, then concentrations of glucose (**j**), prostaglandin E2 (PGE2) (**k**) and lactate (**l**) were analyzed. For all panels, error bars indicate s.d.. For panel (a-d), (g) and (j-l), n = 3 biological replicates, average of n=5 technical replicates for each biological replicate was used. The two-tailed unpaired student t-test was used.

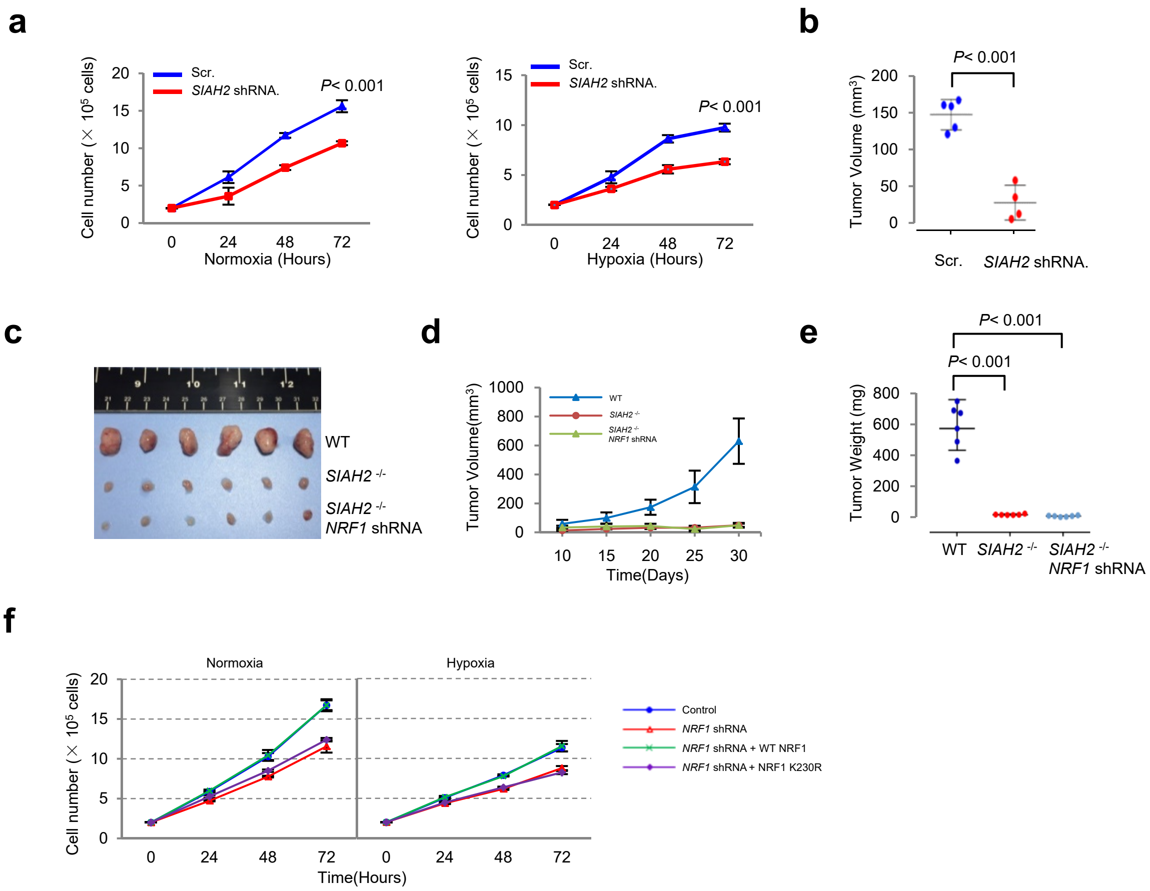

Supplementary Figure 7 SIAH2-NRF1 axis regulates tumor growth and cell proliferation.(**a**) Growth curves of wild-type and SIAH2-knockdown MDA-MB-231 cells cultured under normoxia and hypoxia. (**b**) Volumes of wild-type and SIAH2-knockdown xenograft tumors. (**c**-**e**) Images (**c**), growth curves (**d**) and weights (**e**) of xenograft tumors derived from MDA-MB-231 cells with the indicated modifications. Tumors were established in mice by subcutaneous injection of cells. (**f**) Stable NRF1-knockdown MDA-MB-231 cells reconstituted with wild-type NRF1 or the NRF1-K230R mutant, together with mock and NRF1-knockdown MDA-MB-231 cells, were cultured under normoxia or hypoxia, cell numbers were calculated at the indicated time points. For all panels, error bars indicate s.d.. For panel (a) and (f) n = 3 biological replicates, average of n=5 technical replicates for each biological replicate was used. For panel (b), n = 5 mice per group. For panel (c-e), n = 6 mice per group. The two-tailed unpaired student t-test was used.

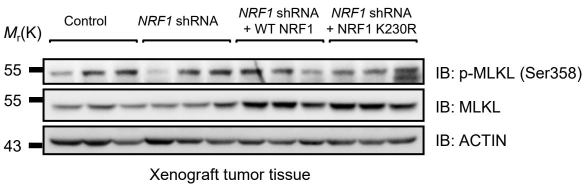

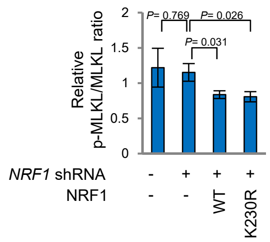

Supplementary Figure 8 NRF1-K230R-induced tissue damage is not due to MLKL-mediated necroptosis. Three groups of fresh frozen tissues from indicated xenograft tumors were analyzed by western blotting with the anti-p-MLKL (Ser358) and anti-MLKL antibodies. Quantification of the ratio of p-MLKL versus to total MLKL protein levels was shown below. n = 3 biological replicates. The two-tailed unpaired student t-test was used to compare data.

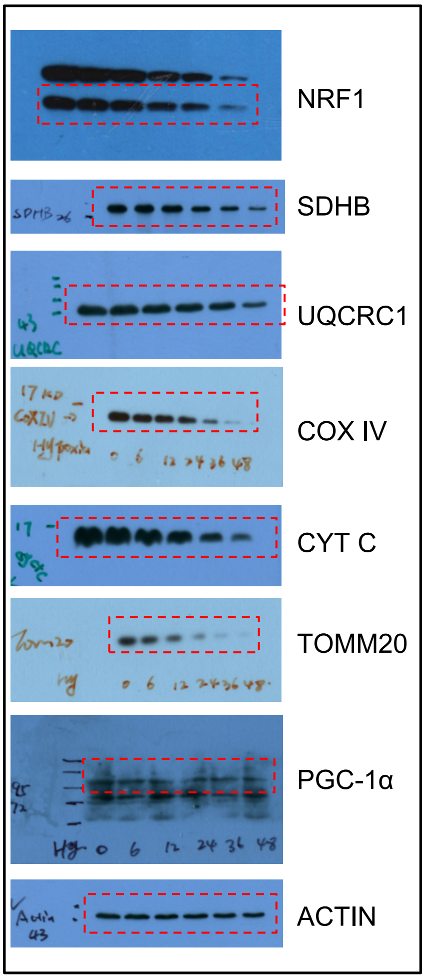

Figure 2a

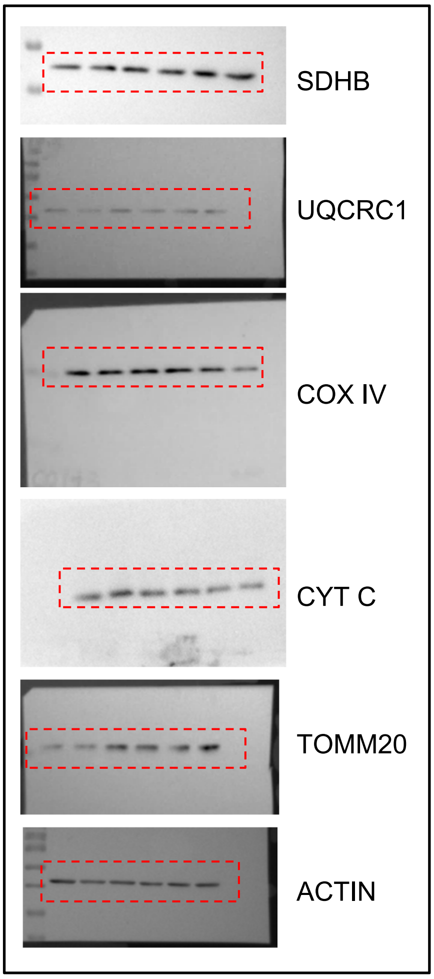

Figure 2c

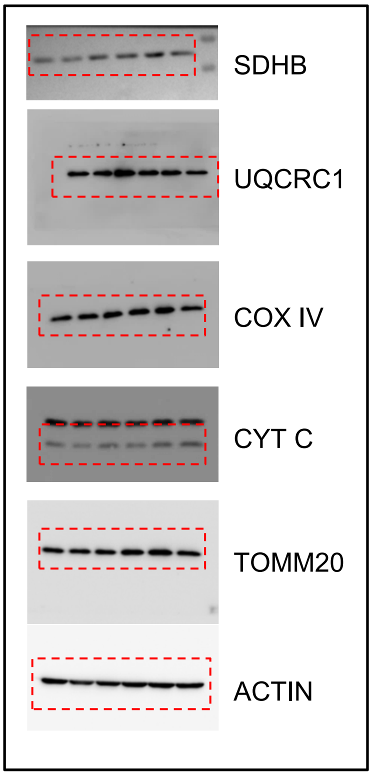

Figure 2d

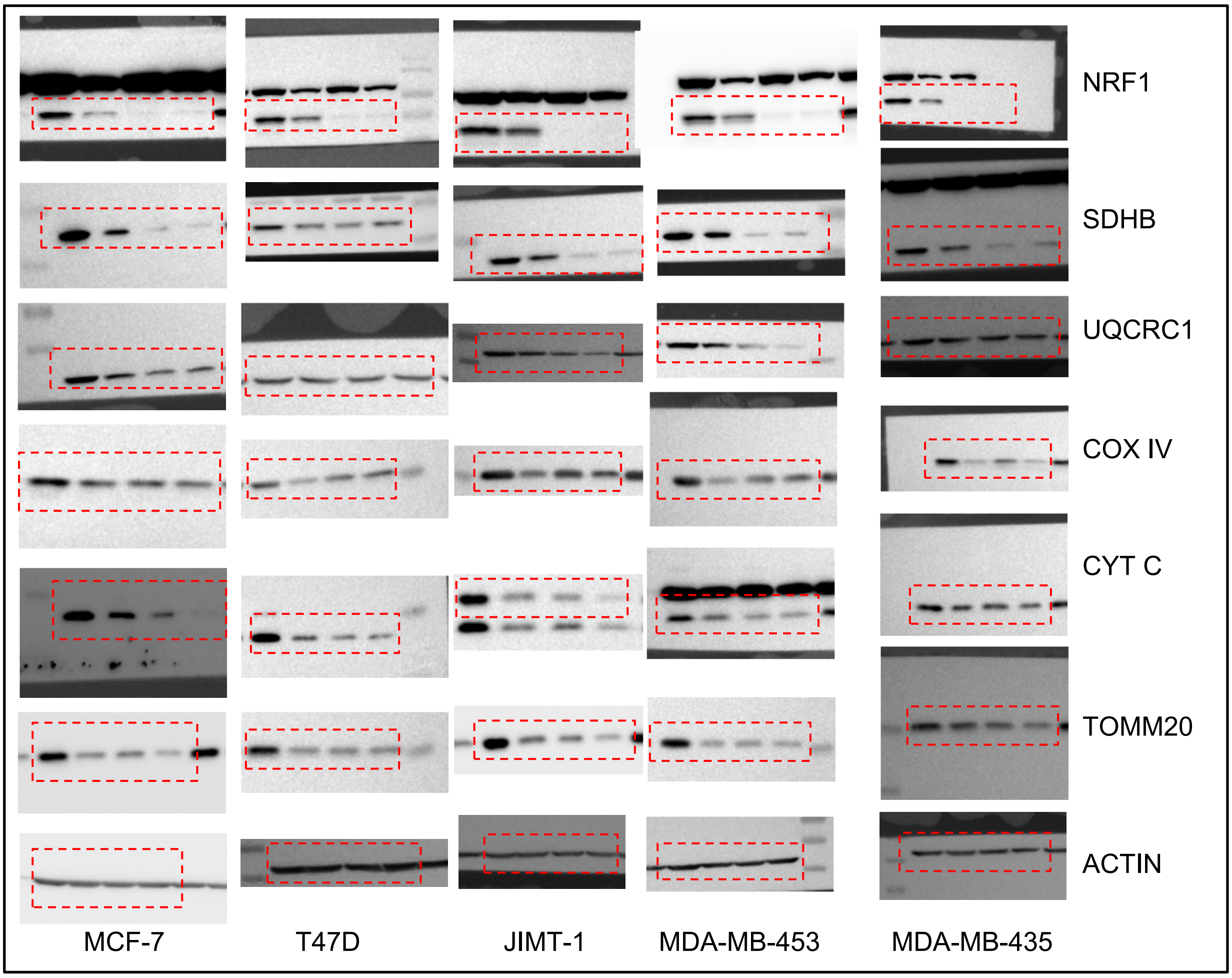

Figure 2e

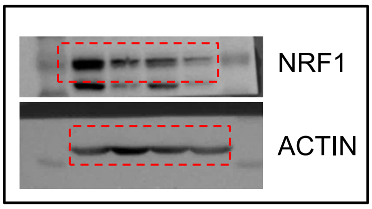

Figure 3a

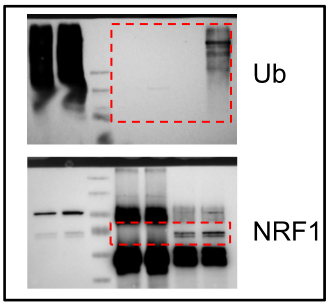

Figure 3b

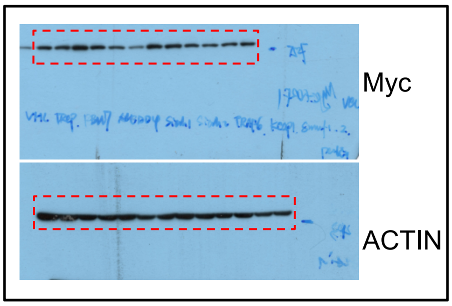

Figure 3c

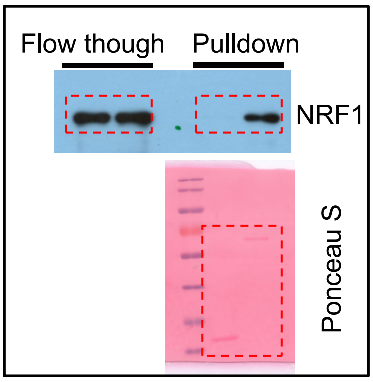

Figure 3d

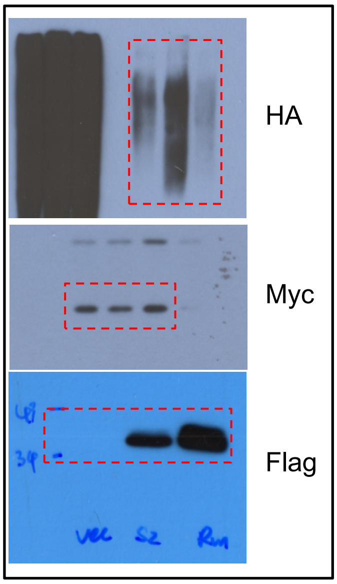

Figure 3e

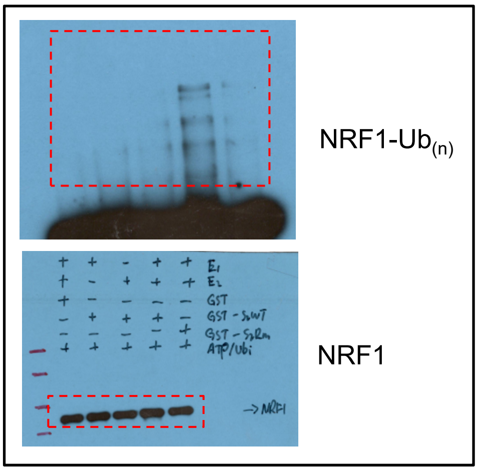

Figure 3f

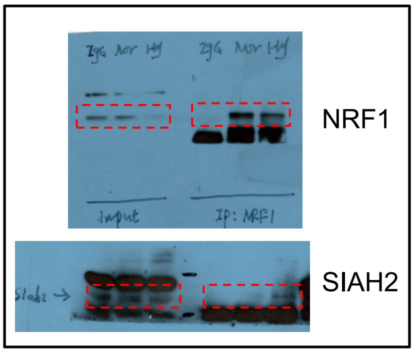

Figure 3g

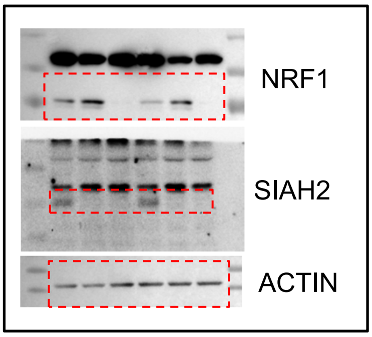

Figure 3h

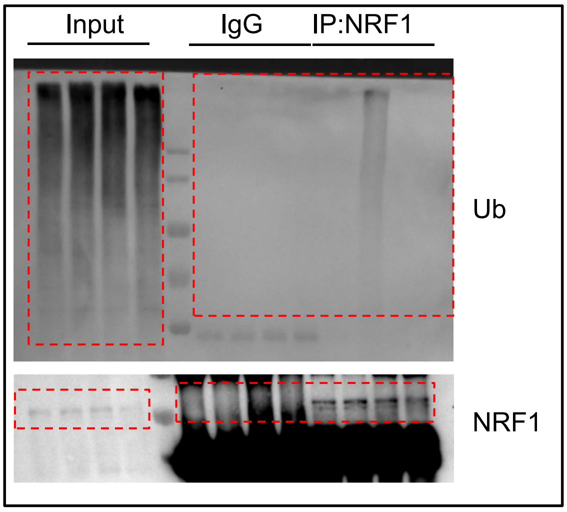

Figure 3i

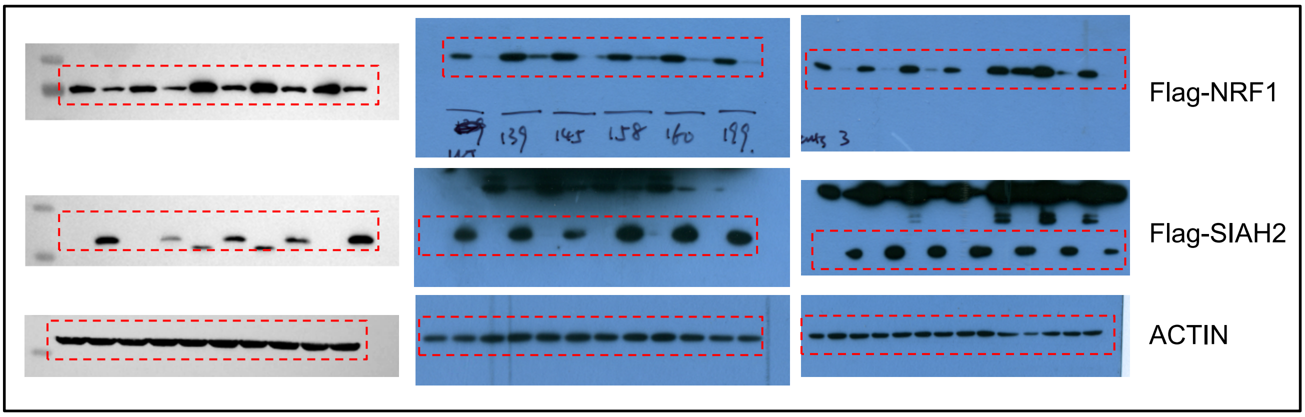

Figure 4a

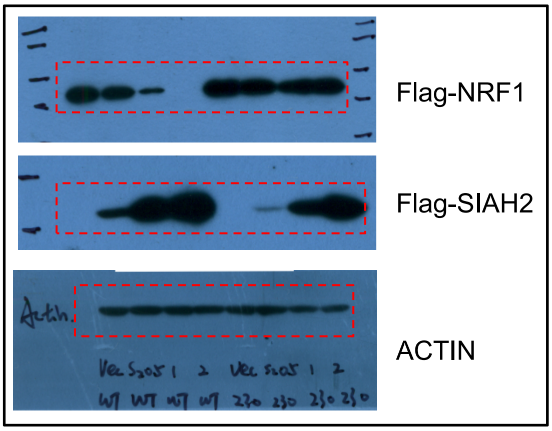

Figure 4b

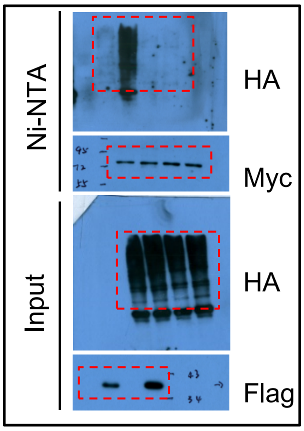

Figure 4c

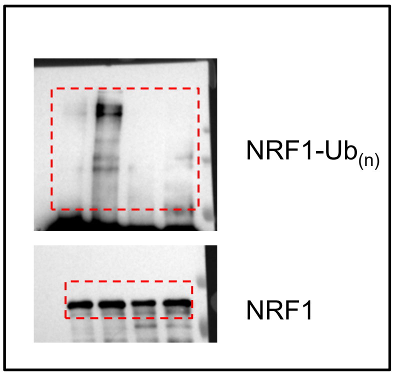

Figure 4d

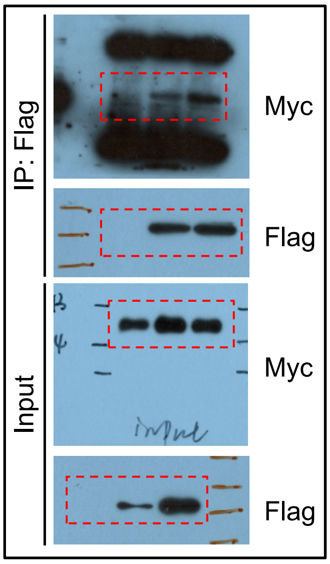

Figure 4e

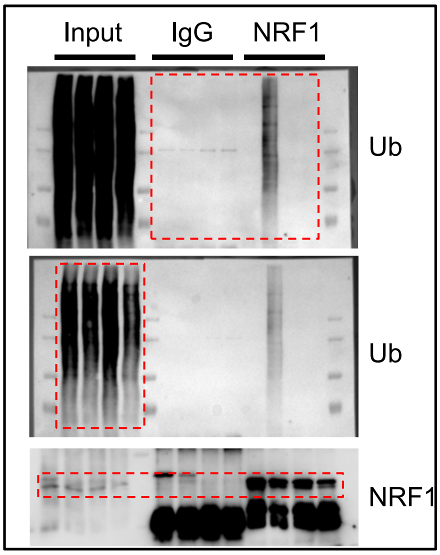

Figure 4f

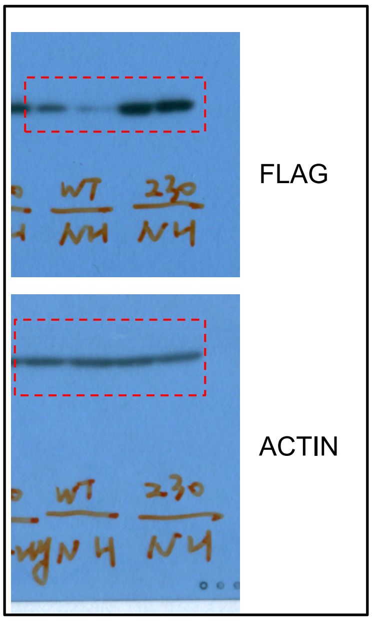

Figure 4g

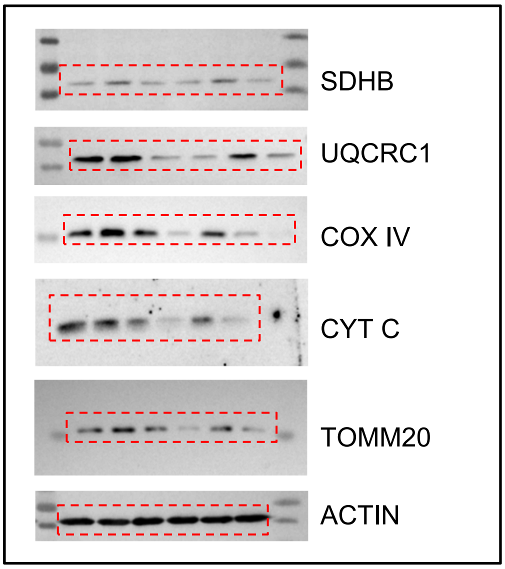

Figure 5a

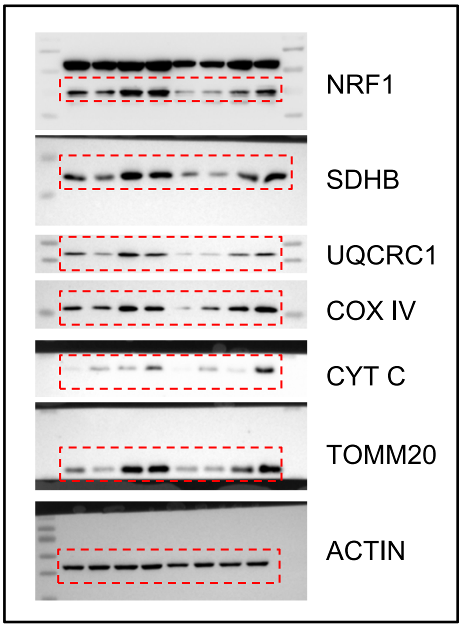

Figure 5c

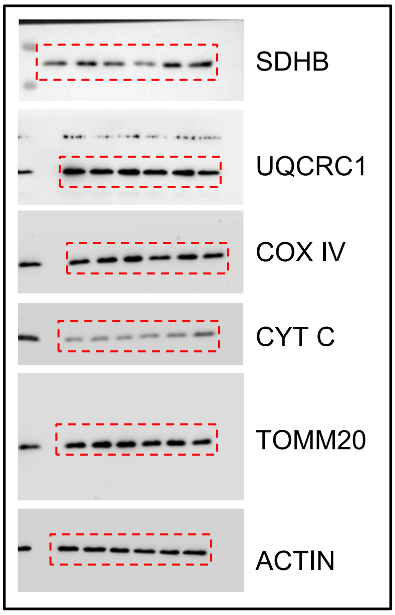

Figure 5e

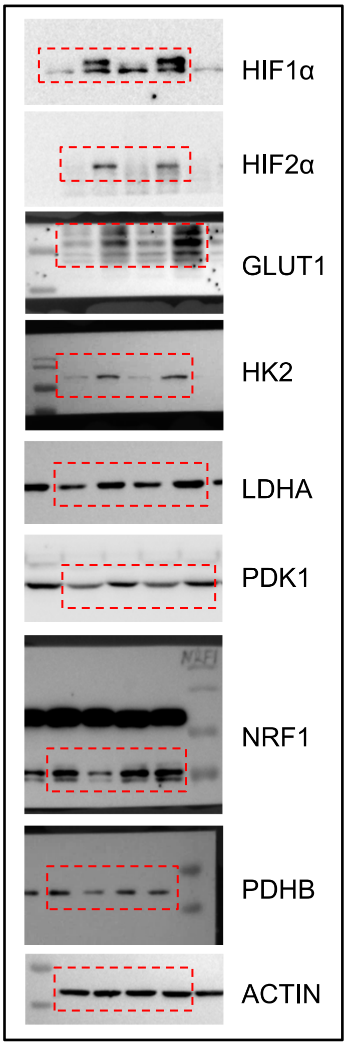

Figure 6k

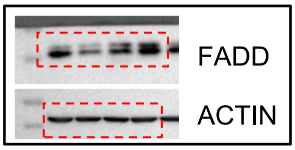

Figure 8g

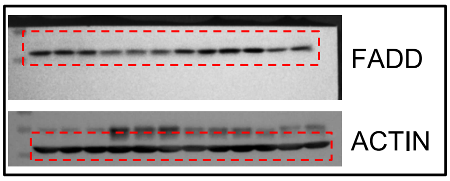

Figure 8h

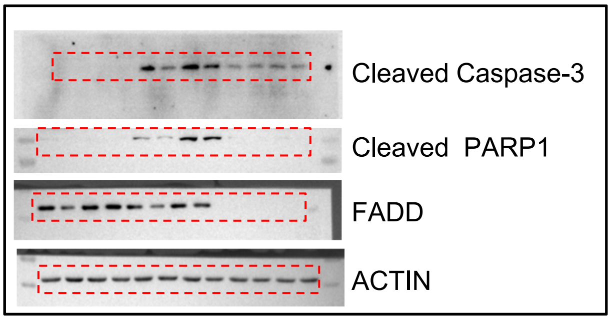

Figure 8i

Supplementary Figure 9 Uncropped images of key blots.

Supplementary Table 1 Prohibitin is reduced in breast cancer

IHC criteria		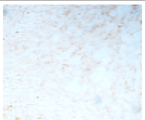	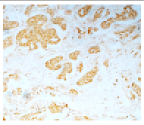	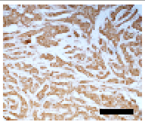
Prohibitin Staining	Total	**+**	**++**	**+++**
Normal breast tissue	27	6 (22.2%)	14 (51.9%)	7 (25.9%)
Breast tumor tissue	158	75 (47.5%)	67 (42.4%)	16 (10.1%)

P= 0.015

Supplementary Table 1 Prohibitin is reduced in breast cancer. Scale bars, 200 μm. Brown color indicates positive immune reaction. Statistical significance was determined by χ2 test.

Supplementary Table 2 Patient characteristics based on Prohibitin expression

		Prohibitin Staining Intensity			
Variables	Total	+	++	+++	*P*-value
Age					0.965
>50 years	67	31 (46.3%)	29 (43.3%)	7 (10.4%)	
≤ 50 years	91	44 (48.4%)	38 (41.7%)	9 (9.90%)	
Types					0.036
invasive ductal carcinoma (IDC)	79	31 (39.2%)	36 (45.6%)	12 (15.2%)	
invasive lobular carcinoma (ILC)	79	44 (55.7%)	31 (39.2%)	4 (5.10%)	
T Stage					0.002
T1	11	2 (18.2%)	5 (45.4%)	4 (36.4%)	
T2	112	48 (42.9%)	52 (46.4%)	12 (10.7%)	
T3	17	11 (64.7%)	6 (35.3%)	0 (0.00%)	
T4	18	14 (78.8%)	4 (22.2%)	0 (0.00%)	
AJCC stage					0.025
I/IIa	73	27 (37.0%)	35 (47.9%)	11 (15.1%)	
IIb/III	85	48 (56.5%)	32 (37.6%)	5 (5.90%)	
Lymph node metastasis					0.848
N0	92	42 (45.7%)	40 (43.5%)	10 (10.9%)	
N1/2	66	33 (50.0%)	27 (40.9%)	6 (9.10%)	
ER positivity					0.570
negative	76	40 (52.6%)	29 (38.2%)	7 (9.20%)	
1+	34	14 (41.2%)	18 (52.9%)	2 (5.90%)	
2+	28	14 (50.0%)	10 (35.7%)	4 (14.3%)	
3+	20	7 (35.0%)	10 (50.0%)	3 (15.0%)	
HER2 positivity					0.006
negative	121	62 (51.2%)	49 (40.5%)	10 (8.30%)	
1+	14	6 (42.9%)	8 (57.1%)	0 (0.00%)	
2+	15	6 (40.0%)	7 (46.7%)	2 (13.3%)	
3+	8	1 (12.5%)	3 (37.5%)	4 (50.0%)	
PR positivity					0.118
negative	91	51 (56.0%)	32 (35.2%)	8 (8.80%)	
1+	30	10 (33.3%)	18 (60.0%)	2 (6.70%)	
2+	30	12 (40.0%)	14 (46.7%)	4 (13.3%)	
3+	7	2 (28.6%)	3 (42.8%)	2 (28.6%)	
triple negative					0.171
Yes	48	28 (58.3%)	17 (35.4%)	3 (6.30%)	
No	110	47 (42.7%)	50 (45.5%)	13 (11.8%)	

Supplementary Table 3 SIAH2 is increased in breast cancer

IHC criteria

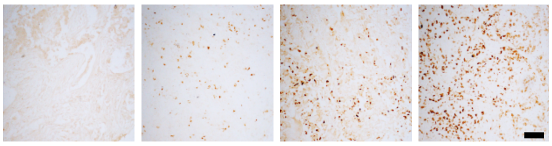

SIAH2 expression	Negative	+	++	+++	Total
	Low		Moderate	high	
Normal breast tissue	16 (59.3%)	8 (29.6%)	3 (11.1%)	0 (0%)	27
Total	24 (88.9%)		3 (11.1%)	0 (0%)	
Breast Tumor	41 (25.6%)	61 (38.1%)	39 (24.4%)	19 (11.9%)	160
Total	102 (63.8%)		39 (24.4%)	19 (11.9%)	

P=0.003

Supplementary Table 3 SIAH2 is increased in breast cancer. Scale bars, 100 µm. Brown color indicates positive immune reaction. Statistical significance was determined by χ^2 test.

Supplementary Table 4 NRF1 is reduced in breast cancer

IHC criteria

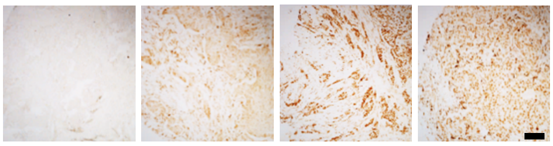

NRF1 expression	Negative	+	++	+++	Total
	Low		Moderate	high	
Normal breast tissue	0 (0%)	3 (11.1%)	14 (51.9%)	10 (37%)	27
Total	3 (11.1%)		14 (51.9%)	10 (37%)	
Breast Tumor	17 (10.6%)	79 (49.4%)	42 (26.3%)	22 (13.7%)	160
Total	96 (60.0%)		42(26.3%)	22 (13.7%)	

P< 0.001

Supplementary Table 4 NRF1 is reduced in breast cancer. Scale bars, 100 μm. Brown color indicates positive immune reaction. Statistical significance was determined by χ2 test.

Supplementary Table 5 Patient characteristics based on NRF1 expression

		NRF1 expression		
Variables	Total	Negative/+	++/+++	*P*-value
Age				0.948
>50 years	67	40 (59.7%)	27 (40.3%)	
≤ 50 years	93	56 (60.2%)	37 (39.8%)	
Types				0.517
IDC	80	46 (57.5%)	34 (42.5%)	
ILC	80	50 (62.5%)	30 (37.5%)	
T Stage				0.030
T1/T2	125	69 (55.2%)	56 (44.8%)	
T3/T4	35	26 (74.3%)	9 (25.7%)	
AJCC stage				0.271
I/IIa	74	41 (55.4%)	33 (44.6%)	
IIb/III	86	55 (64.0%)	31 (36.0%)	
Lymph node metastasis				0.948
N0	93	56 (60.2%)	37 (39.8%)	
N1/2	67	40 (59.7%)	27 (40.3%)	
